# Outcomes Following Transoral Laser Microsurgery for T1b and T2a Glottic Squamous Cell Carcinoma With and Without Anterior Commissure Involvement: A Retrospective Chart Review

**DOI:** 10.1177/19160216251348424

**Published:** 2025-06-18

**Authors:** Depak Patel, Victoria Taylor, Colin MacKay, Chrisje den Besten, Matthew H. Rigby, Martin Corsten, Timothy Brown, Jonathan Trites, S. Mark Taylor

**Affiliations:** 1 Division of Otolaryngology—Head and Neck Surgery, Dalhousie University, Halifax, NS, Canada; 2 Faculty of Medicine, Dalhousie University, Halifax, NS, Canada; 3 Department of Otolaryngology—Head and Neck Surgery, Haaglanden MC, The Hague, The Netherlands; 4 Department of Otolaryngology—Head and Neck Surgery, AdventHealth Medical Group, Daytona Beach, FL, USA

**Keywords:** transoral laser microsurgery, glottic cancer, laryngeal cancer

## Abstract

**Importance:**

There is a limited understanding of anterior commissure (AC) involvement in glottic squamous cell carcinoma (SCC), particularly when comparing T1b, T2a with AC involvement (T2AC), and T2a without AC involvement (T2noAC).

**Objective:**

The aim of this study was to compare oncological and functional outcomes in T1b, T2AC, and T2noAC glottic SCC following transoral laser microsurgery (TLM).

**Design:**

Retrospective chart review.

**Setting:**

The Queen Elizabeth II Health Science Centre (Halifax, Nova Scotia) from January 1, 2002, to December 31, 2022.

**Intervention and Exposures:**

A retrospective chart review was completed using prospectively-collected data for patients treated with TLM for T1b and T2a glottic SCC. Exclusion criteria included previous treatment for a laryngeal cancer and T2b glottic SCC.

**Main Outcome Measures:**

Oncological outcomes were assessed using margin status, local control (LC), disease-specific survival (DSS), overall survival (OS), and laryngeal preservation (LP). Functional outcomes were measured using the abbreviated Voice Handicap Index-10 (VHI-10).

**Results:**

In total, 117 patients were included (T1b = 46, T2AC = 53, T2noAC = 18). Positive margins were higher in the T2AC group (15.1%) than in T1b (4.3%) and T2noAC (5.6%; *P* = .208). At 5 years, there were no significant differences in LC (T1b = 80.8%, T2AC = 70.3%, T2noAC = 76.2%; *P* = .26), DSS (T1b = 100%, T2AC = 90.2%, T2noAC = 93.8%; *P* = .45), OS (T1b = 88.3%, T2AC = 76.1%, T2noAC = 93.8%; *P* = .69), or LP (T1b = 94.3%, T2AC = 92.1%, T2noAC = 94.4%; *P* = .74). Significant improvements in VHI-10 scores from the pre- to postoperative period were only noted in the T1b cohort, at the 6 months (*P* = .017) and the 12 months (*P* = .00143).

**Conclusions:**

No significant differences in both oncological and functional outcomes were noted between T1b, T2AC, and T2noAC glottic SCCs. Further stratifying based on the degree and pattern of AC involvement with larger sample sizes may provide important prognostic factors.

**Relevance:**

This study highlights that T2 glottic SCCs with normal vocal fold mobility are a heterogenous group, and it may be beneficial to further stratify these cancers according to AC involvement, particularly when considering TLM.

## Graphical abstract

**Graphical Abstract:**
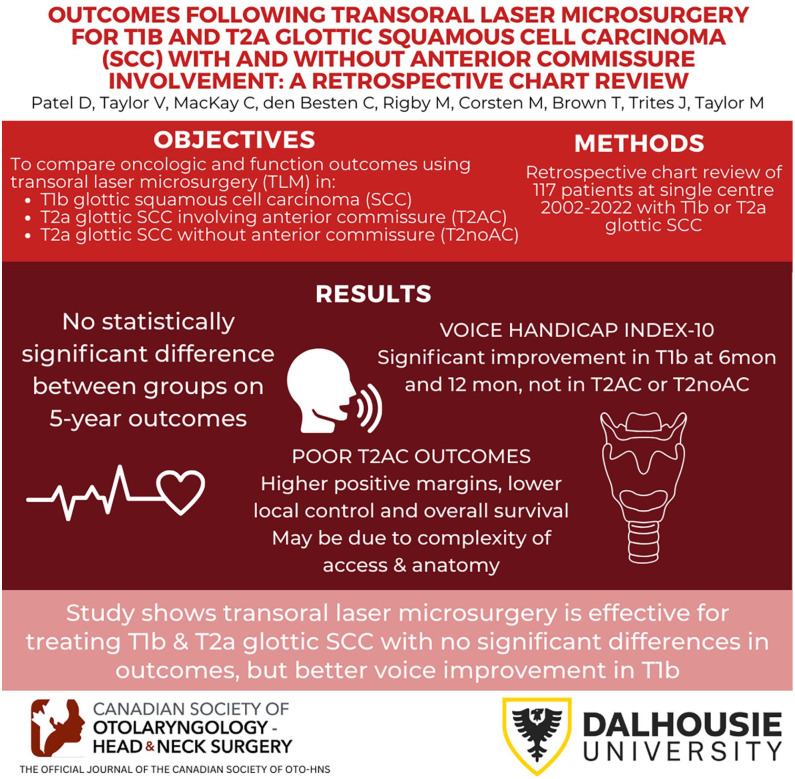

## Key Message

- Higher rates of positive margins in glottic squamous cell carcinoma (SCC) with anterior commissure (AC) involvement highlight potential challenges in achieving oncological control.
- Transoral laser microsurgery remains a valuable and minimally-invasive treatment for T1b/T2a glottic SCC.
- Further stratification and characterization of AC involvement may identify critical prognostic factors.

## Introduction

Laryngeal cancer, specifically squamous cell carcinoma (SCC), is among the most common malignancies in otolaryngology, accounting for 30% to 40% of all head and neck cancers.^1^ Glottic involvement is present in ~65% of laryngeal SCC cases.^2^ The anterior commissure (AC) comprises the anterior area of the glottis where the 2 vocal folds attach into the thyroid cartilage.^3^ AC involvement is present in nearly 20% of all glottic tumors.^4^ Several authors suspect that the fibrous tissue of the AC tendon prevents tumor spread due to its mechanical barrier^5,6^; conversely, others believe that this anatomical dehiscence in thyroid perichondrium facilitates tumor spread.^7^

As glottic SCCs progress from T1 through to T4, both disease-free survival and overall survival (OS) decrease.^8^ The current American Joint Committee on Cancer (AJCC) eighth edition staging system for glottic cancer upstages a T1a glottic tumor to a T1b if the AC is involved. This is secondary to research demonstrating AC involvement to be a negative prognostic factor in T1 glottic cancers.^9^ T2 glottic tumors involve extension from the glottis to 1 or more subsites of the larynx: the false vocal cords, epiglottis, or subglottis. The AJCC does not, however, differentiate between T2 tumors with and without AC involvement.

Transoral laser microsurgery (TLM) for early glottic cancer was originally described by Strong and Jako^10^ and popularized by Steiner.^11^ Although T1a tumors are almost universally treated with TLM, T1b, and T2 tumors still remain a subject of treatment debate among head and neck surgeons.^12^

Previous research at our institution has shown that when T2 glottic tumors are stratified by normal vocal fold mobility (T2a) and hypomobility (T2b), local control (LC) and voice outcomes are significantly lower in the T2b group.^13^ Ultimately, this research demonstrated that T2 tumors with hypomobile vocal folds behave more aggressively, comparable to T3 glottic SCC, than those with normal vocal fold mobility. However, there is currently limited research specifically assessing the role of AC involvement on voice and oncological outcomes in T2a tumors. The aim of this study was to compare oncological and functional outcomes of T1b, T2a tumors with AC involvement (T2AC), and T2 tumors without AC involvement (T2noAC). We hypothesized that functional and oncological outcomes would be worse in the T2AC group than in both the T1b and T2noAC groups.

## Methods

This study received ethics approval through the Nova Scotia Health Research Ethics Board (1020643).

### Design and Participants

A retrospective chart review of prospectively-collected data for patients undergoing TLM for T1b and T2a glottic SCC at The Queen Elizabeth II Health Science Centre (Halifax, Nova Scotia) from January 1, 2002, to December 31, 2022, was conducted. Given the retrospective nature of this study and the use of de-identified patient data, informed consent was waived. TLMs were performed by 3 otolaryngologists fellowship-trained in head and neck surgical oncology. Patients were excluded if they had T2b glottic SCC or they had previously been treated with radiation, chemotherapy, or surgery (including TLM) for laryngeal cancer. Tumor staging was completed using the appropriate AJCC edition (sixth, seventh, or eighth edition) at the time of diagnosis.

### Outcomes and Measures

Patient clinicodemographic information was collected, including age, sex, smoking history, time of follow-up, and node status (positive or negative). Oncological outcomes were evaluated using LC, margin status, disease specific survival (DSS), OS, and laryngeal preservation (LP) up to 5 years after TLM. Margin analysis was conducted on surrounding tissue after the primary specimen was removed. Frozen section analysis was not routinely performed.

Functional outcomes were assessed using the abbreviated Voice Handicap Index-10 (VHI-10) questionnaire. The VHI is a reliable, valid self-report questionnaire that assesses patients’ perceptions of vocal handicap.^14^ An abbreviated 10-item version of the VHI questionnaire, the VHI-10, was used in this study. The 10 items are rated on a 5-Point Likert scale from 0 (never) to 4 (always), with higher scores indicating higher levels of perceived vocal handicap. Scores >11 are considered abnormal.^15^ The VHI-10 was administered to patients in the preoperative period as well as the postoperative period at the 3rd, 6th, and 12th month follow-up.^16^

### Statistical Analysis

Data analyses were completed using the R Statistical Software (R Core Team 2022; version 4.2.2). Demographic information was analyzed using univariate statistics. Age and time of follow-up were both continuous variables, while smoking history, node status, and sex were categorical variables. OS, DSS, LC, and LP were calculated using Kaplan-Meier survival curves. Fisher’s exact tests were used to compare demographic information and oncological outcomes between groups. Survival comparisons between groups were completed using log-rank tests. VHI-10 scores were compared between the preoperative period and postoperative follow-ups using paired t-tests, and *P* values were adjusted for multiple tests.

## Results

### Patient Demographics

In total, 117 patients were included in the study. The majority of patients were male (88.9%), current or previous smokers (88.9%), and the most common tumor stage was T2AC (45.3%). Mean follow-up was 54.4 months (range 4-220) for the T1b group, 52.6 months (range 5-157) for T2AC, and 71.3 months (range 7-144) for T2noAC. One patient in the T2AC group had regional disease on presentation (node positive; N2b disease) requiring a neck dissection simultaneously as their primary tumor surgery (Table 1).

**Table 1.** Patient Demographics.

Characteristic	T1b (n = 46)	T2AC (n = 53)	T2noAC (n = 18)	Total (n = 117)	*P* value
Age [mean (SD)]	66.5 (10.5)	65.6 (10.3)	70.1 (9.3)	66.7 (10.2)	.285
Sex [male (%)]	42 (91.3)	48 (90.6)	14 (77.8)	104 (88.9)	.319
Smoking history (%)	42 (35.9)	47 (40.2)	15 (12.8)	104 (88.9)	.513
>10 pack-years	11 (23.9)	18 (34.0)	5 (27.8)	34 (29.1)	
Ex-smoker >10 pack-years	20 (43.5)	23 (43.4)	6 (33.3)	49 (41.9)	
Ex-smoker <10 pack-years	1 (2.2)	0 (0.0)	1 (5.6)	2 (1.7)	
Ex-smoker unknown pack-years	10 (21.7)	6 (11.3)	3 (16.7)	19 (16.2)	
Nonsmoker	3 (6.5)	3 (5.7)	3 (16.7)	9 (7.7)	
Unknown	1 (2.2)	3 (5.7)	0 (0.0)	4 (3.4)	
Alcohol history (%)					.840
<1-7 drinks/wk	27 (58.7)	35 (66.0)	12 (66.7)	74 (63.3)	
<4 drinks/d (male) or <3 drinks/d (female)	1 (2.2)	0 (0.0)	0 (0.0)	1 (0.9)	
>21 drinks/wk	3 (6.5)	4 (7.5)	2 (11.1)	9 (7.7)	
15-21 drinks/wk	3 (6.5)	2 (3.8)	0 (0.0)	5 (4.3)	
7-14 drinks/wk	9 (19.6)	7 (13.2)	4 (22.2)	20 (17.1)	
Unknown	3 (6.5)	5 (9.4)	0 (0.0)	8 (6.8)	
Follow-up [mo (range)]	54.4 (4-220)	52.6 (5-157)	71.3 (7-144)	56.2 (4-220)	.182
Node positive (%)	0 (0.0)	1 (1.9)	0 (0.0)	1 (0.9)	1.000

### Oncological Outcomes

**Figure 1.**
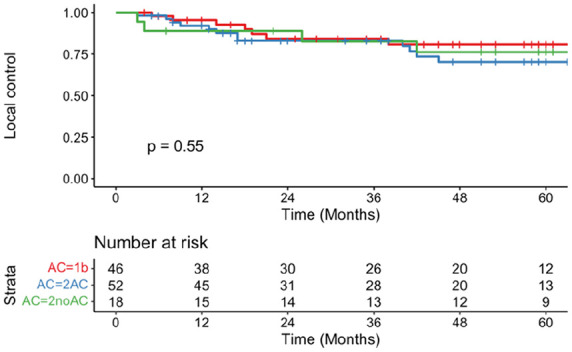
Local control rates of T1b (red), T2AC (blue), and T2noAC (green) over 5 year period.

In total, 21 patients (17.9%) had local recurrences, 3 had locoregional recurrences (2.6%), and 3 (2.6%) had regional recurrences (Table 2). Five-year LC rates for T1b (LC = 80.8%, 95% CI = 68.8-94.9, SE = 8.2), T2AC (LC = 70.3%, 95% CI = 57.1-86.6, SE = 10.7), and T2noAC (LC = 76.2%, 95% CI = 58.2-99.7, SE = 13.7) did not differ significantly (*P* = .55; Figure 1). There was no significant difference in positive margin status between the T1b (4.3%), T2AC (15.1%), and T2noAC (5.6%) groups (*P* = .208). All but 1 patient (due to poor laryngeal exposure) with local recurrence underwent another resection. One patient in the T2AC group had distant disease in the lung.

**Table 2.** Oncological Outcomes.

Outcome	T1b (n = 46)	T2AC (n = 53)	T2noAC (n = 18)	Total (n = 117)	*P* value
Margins (%)					.208
Close	0 (0.0)	1 (1.9)	1 (5.6)	2 (1.7)	
Negative	33 (71.7)	38 (71.7)	13 (72.2)	84 (71.8)	
Positive	2 (4.3)	8 (15.1)	1 (5.6)	11 (9.4)	
Unknown	11 (23.9)	6 (11.3)	3 (16.7)	20 (17.1)	
First recurrence (%)					.672
None	38 (82.6)	38 (71.7)	13 (72.2)	89 (76.1)	
Local	5 (10.9)	11 (20.8)	5 (27.8)	21 (17.9)	
Locoregional	2 (4.3)	1 (1.9)	0 (0.0)	3 (2.6)	
Regional	1 (2.2)	2 (3.8)	0 (0.0)	3 (2.6)	
Regional and metastatic	0 (0.0)	1 (1.9)	0 (0.0)	1 (0.9)	

**Figure 2.**
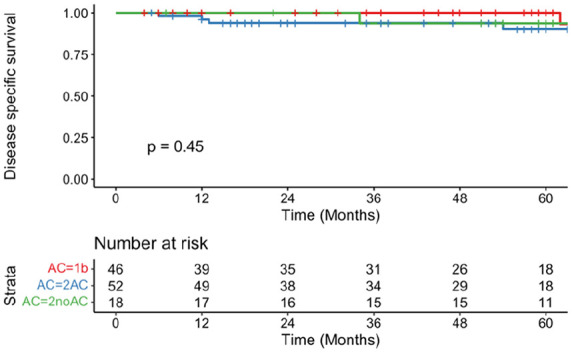
Kaplan-Meier curve representing disease-specific survival of T1b (red), T2AC (blue), and T2noAC (green).

**Figure 3.**
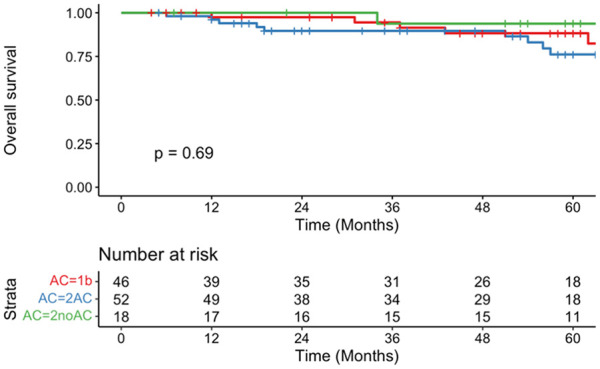
Kaplan-Meier curve representing overall survival of T1b (red), T2AC (blue), and T2noAC (green).

Eleven patients (11.6%) in the T2a group (T2AC = 10, T2noAC = 1) died from their disease within 5 years (T2AC = 90.2%, 95% CI = 81.1-100, SE = 5.4; T2noAC = 93.8%, 95%CI = 82.6-100, SE = 6.5). No T1b patients died from their disease at 5 years (T1b = 100%, 95% CI = 100-100, SE = 0). DSS did not differ significantly between the 3 groups (*P* = .45; Figure 2). OS at 5 years was not significantly different between groups (T1b = 88.3%, 95% CI = 78.1-99.8, SE = 6.3; T2AC = 76.1%, 95% CI = 63.2-91.8, SE = 9.5; T2noAC = 93.8%, 95% CI = 82.6-100, SE = 6.5; *P* = .69; Figure 3).

Seven patients required tracheostomies, 4 of whom did not require a total laryngectomy (TL). One patient presented with airway obstruction but did not require a TL, 2 were due to a large or recurrent second primary, and 1 for postradiation scarring (Table 3). The Kaplan-Meier LP rate at 5 years was 91.1% (T1b = 94.3%, 95% CI = 86.7-100, SE = 4.3; T2AC = 92.1%, 95% CI = 83.6-100, SE = 4.9; T2noAC = 94.4%, 95% CI = 84.4-100, SE = 5.7). Eight patients failed organ preservation requiring a laryngectomy. Three of the 8 laryngectomies were salvage surgeries following radiation/chemoradiation therapy, 2 due to large local recurrences, 1 due to multiple recurrences treated with TLM, and 1 after 2 regional recurrences followed by a local recurrence. Seven patients required gastrotomy tubes (6.0%).

**Table 3.** Swallowing and Respiratory Outcomes.

Outcome	T1b (n = 46)	T2AC (n = 53)	T2noAC (n = 18)	Total (n = 117)	*P* value
Gastrostomy tube (%)	1 (2.2)	5 (9.4)	1 (5.6)	7 (6.0)	.313
Tracheostomy^a^ (%)	2 (4.3)	2 (3.8)	0 (0.0)	4 (3.4)	1.0
Laryngectomy (%)	4 (8.7)	3 (5.7)	1 (5.6)	8 (6.8)	.883

a Three patients with tracheostomies preceding laryngectomy were not included in the tracheostomy group.

### Functional Outcomes

VHI-10 scores were available for 49 patients (41.9%; T1b = 19, T2AC = 19, T2noAC = 11).

**Figure 4.**
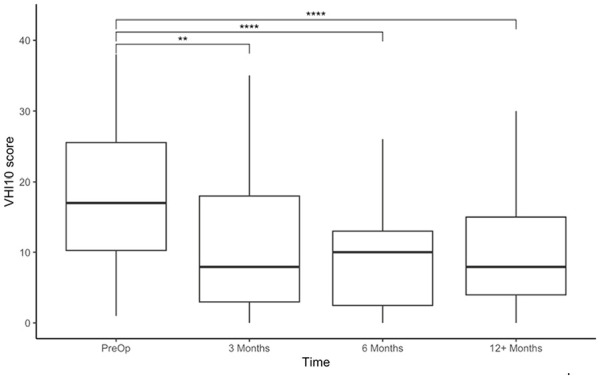
VHI-10 raw scores pre-operatively and post-operatively at 3, 6, and 12 months. VHI-10, Voice Handicap Index-10. **P < .01. ****P < .0001.

**Figure 5.**
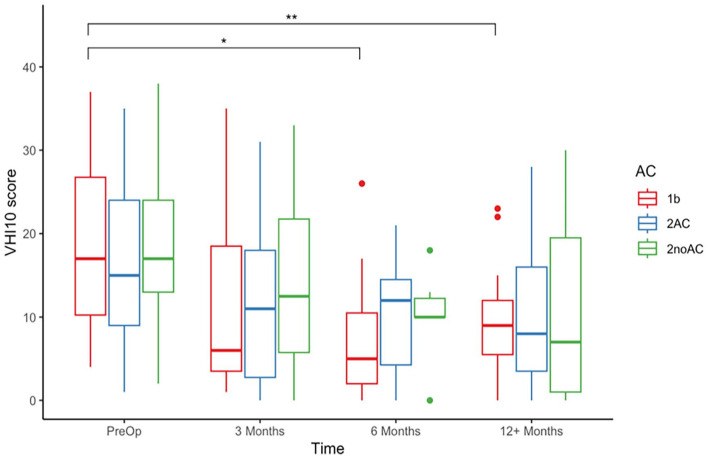
VHI-10 raw scores pre-operatively and post-operatively at 3, 6, and 12 months stratified by anterior commissure involvement for T1b (red), T2AC (blue), and T2noAC (green). VHI-10, Voice Handicap Index-10. *P < .05. **P < .01.

Statistically significant improvements in VHI-10 scores were shown for the whole cohort at all 3 post-operative time points when compared to pre-operatively (Figure 4). When the cohort was stratified by stage and AC involvement, the only statistically significant improvements were seen for T1b patients at 6 months post-operatively (*P* = .017) and at 12 months postoperatively (*P* = .00143; Figure 5). Mean and median change in VHI-10 score were also assessed, with no significant differences at any of the post-operative time points for any of the subgroups.

## Discussion

The aim of this study was to compare functional and oncological outcomes between T1b and T2a glottic SCCs with (T2AC) and without (T2noAC) AC involvement. The significance of AC involvement in early stage glottic SCC has been the subject of debate with regard to oncological outcomes,^17^ and has been a topic of interest for many researchers.^17-23^ Ideally, treatment for glottic SCCs leads to oncological control with LP, satisfactory phonation and deglutition, and minimal treatment-related side effects. Although radiotherapy is an effective treatment modality for early-laryngeal SCC,^24^ it can be associated with devastating complications such as edema requiring a tracheostomy, aspiration, radiation-induced malignancies, and chondronecrosis. There is a low chance of radiation failure in early glottic cancers^25^; however, disease recurrence in the postradiation setting may result in a TL, often associated with poor wound healing secondary to tissue radiation. On the other hand, disease recurrences after TLM may be re-resected, avoiding the many complications linked to radiation exposure, while reducing the likelihood of a major head and neck surgery (ie, laryngectomy).

Research assessing functional outcomes in T2 glottic carcinoma treated with TLM is lacking.^26^ Significant VHI-10 improvements have been noted in T2a tumors (6.6 from 21.3) compared with those in T2b tumors (21.3 from 21.4; *P* < .001) following TLM.^13^ Generally, voice outcomes among individuals with extended T1 and limited T2 glottic cancer are good, with low self-reported voice impairment (VHI = 23.3-24.5).^27^ This is the first study to compare pre- and post-TLM voice outcomes between T1b and T2 tumors with normal vocal fold mobility (T2a) with and without AC involvement. Our study found no significant difference in mean VHI-10 improvements at 12 months after TLM between T1b, T2AC, and T2noAC glottic SCCs. However, VHI-10 data were missing for a significant percentage of the postoperative patients; therefore, larger sample sizes are required for better differentiation of functional outcomes between groups.

Although not statistically significant, higher rates of 5 year DSS, OS, and LC were noted in patients with T2noAC than in those with T2AC. Furthermore, there was a nonsignificant, higher OS for patients with T2noAC compared with those with T1b tumors. This demonstrates that glottic tumors with AC involvement, such as T1b and T2AC, may present greater treatment challenges than those without AC involvement (T2noAC), especially given their anatomic vulnerability. A larger sample size could provide further information. T2AC should be considered a separate disease entity to the TLM head and neck surgeon. Treatment decisions should, however, be made on a case-by-case basis until further research provides clearer guidelines.

Although not statistically significant, in this study positive margin rates were higher among T2AC (15.1%) patients than among those with T2noAC (5.6%), likely due to poor exposure in the operating room, leading to difficulty completely excising the tumor with adequate grossly-clear margins. Additionally, evaluating margins following TLM may be difficult due to laser coagulation artifacts and orientation issues.^28^ Although significant associations were noted between positive margins and local disease recurrence, prompt re-resection resulted in no significant difference in DSS or OS at 5 years. Numerous authors have commented on this phenomenon as a strength of TLM all while preventing exposure to radiation therapy.^11,29^

LP in our study suggested a nonsignificant trend in favor of T2noAC (94.4%) and T1b (94.3%) compared with T2AC (92.1%) when treated with TLM. This is comparable to a recent systematic review that demonstrated 88.8% LP in patients with T2 glottic cancers treated with TLM versus 79% in patients treated with radiotherapy.^26^ The study also demonstrated that LP and oncological outcomes were not significantly different between T2 patients with and without AC involvement when treated with TLM.^26^ Additionally, a study involving 50 patients with T1b, and 103 patients with T2a glottic SCCs treated with TLM found no difference in LC, LP, and OS between the groups.^29^ When the T2 tumors were compared specifically based on AC involvement, there was no significant difference in OS at the 5th year in the AC group and identical DSS at the 5th year. Considering the results are mixed, there may be other factors worth considering when treating patients with T1b/T2 glottic SCCs with AC involvement.

Although the literature is mixed, a review by Hendriksma et al determined that when detailed classifications of AC are utilized, there are clear associations between AC involvement and oncological outcomes.^18^ Rucci et al proposed a classification system for AC involvement that involves 4 subgroups: AC0 (no AC involvement), AC1 (AC involvement on 1 side of the midline), AC2 (AC involvement that crosses midline at only 1 part of the vertical plane), and AC3 (whole AC involvement, on both sides of the midline).^30^ A recent study involving 45 patients with T1b glottic carcinoma found that both stage AC3 and elevated vertical extension ratio were statistically-poor prognostic factors; however, TNM staging was not.^20^ Although the current study did not detect statistically-significant differences in oncological outcomes between glottic SCCs with and without AC involvement, AC involvement was not quantified. The lack of quantification of AC involvement may have ultimately led to smaller differences between groups.

This study has several limitations. Of note, oncological and functional outcomes were not compared between the 3 surgeons, and surgeons’ experience with TLM was not explicitly explored. However, previous research has demonstrated that the level of experience with TLM does not influence rates of complications nor DSS for early tumors (Tis/T1/T2).^31^ Future research may seek to understand whether outcomes differ between clinicians with varying levels of experience specifically when the AC is involved. Based on the experience from surgeons at our center, there is a significant learning curve for TLM, which is notably larger for glottic cancers involving the AC. To add, surgical techniques used by the surgeons were not classified in this study (eg, classification of cordectomy); however, it is likely that surgical practices differ between institutions. This needs to be accounted for when considering the results of this study. Additionally, the retrospective design of this study limited our ability to stratify cases based on the degree of AC involvement and to control for confounding variables. An additional limitation, as previously noted, was the significant number of missing postoperative VHI-10 data. Due to the retrospective nature of the study, we were unable to fill in these gaps. Although this study found no difference in functional outcomes between T1b, T2AC, and T2noAC glottic cancers using the VHI-10, this study did not use objective data including acoustics and aerodynamics nor perceptual evaluation of voice to assess functional outcomes. Future research should consider integrating further objective voice data for a more comprehensive assessment of voice outcomes.

## Conclusion

TLM is a minimally-invasive, effective treatment option for T1b and T2a glottic SCC. T2a glottic SCC with normal vocal fold mobility is a heterogenous group that should be further stratified according to AC involvement, particularly when considering TLM. While the results of this study were not statistically significant, 5 year DSS, OS, and LC rates were higher in patients with T2noAC than in those with T2AC. Future studies with larger sample sizes may benefit from further quantifying AC based on the level of involvement to better understand how AC involvement impacts functional and oncological outcomes for early-stage glottic carcinoma.
